# Whole slide imaging for teleconsultation and clinical use

**DOI:** 10.4103/2153-3539.65342

**Published:** 2010-07-13

**Authors:** Bryan Dangott, Anil Parwani

**Affiliations:** Department of Pathology, Shadyside Hospital, University of Pittsburgh Medical Center, 5150 Centre Ave., POB 2, Rm 201, Pittsburgh, PA 15232, USA

A thorough comparison of digital slide diagnosis with glass slide diagnosis is a critical hurdle for clinical use of whole slide scanners. The study by Wilbur *et al*. titled “Whole-slide imaging digital pathology as a platform for teleconsultation” examined the issue by specifically selecting difficult cases representing a broad spectrum of pathology from a variety of organ systems. The cases were characteristic of material that would often be referred for consultation. They then directly compared the Whole Slide Imaging Interpretation (WSII) with the Glass Slide Interpretation (GSI). A reference diagnosis was rendered on glass slides prior to sending the case for WSII and GSI consultations. Both WSII and GSI were performed at a third party lab by separate pathologists who were given limited clinical information. Thus, the consulting pathologists were forced to make a diagnosis on morphology alone without access to gross examinations, reference diagnosis, or referring physicians. A Zeiss Mirax Desk Scanner was used to create the WSI. However, the scanning magnification was not specified. The authors found an overall concordance of 91% between WSII and GSI, which is in line with other studies comparing these modalities.[1] They further analyzed the results by categorizing diagnoses into neoplastic and non-neoplastic groups. The neoplastic group performed slightly better with 93% concordance in comparison to the non-neoplastic group with 88% concordance, but the difference was not statistically significant. It is important to note that the authors also found three cases (5.7%) where the original glass slide reference diagnosis was discordant with the consultant GSI. Therefore, a built-in glass to glass comparison was performed in the study. It was determined that in these three cases the consultant GSI was most likely to be correct and the original reference diagnosis was incorrect. Assuming that the consultant GSI is the gold standard, WSII had a 90.6% concordance rate while the glass reference diagnosis had a 94.3% concordance rate. This correlates to a 3.7% difference between the consultant WSI concordance and the reference glass concordance to GSI.

## COMMENTS

One of the concerns many pathologists have with digital pathology is the diagnostic capacity of the digital format. By choosing consult level cases for comparison of digital and glass diagnoses, it would seem that this study is a stress test for the diagnostic capacity of digital pathology. One of the key comments in this article was the observation that non-neoplastic conditions were more difficult to diagnose in the digital format. In addition, they made an observation that navigation at high power may have contributed to the difficulty in resolving inflammatory entities. This identifies a potential pitfall of WSI. However, there was no further discussion on whether it was the resolution at high power or the actual navigation that contributed to this difficulty. In practice it is likely to be a combination of both of these factors. Higher magnification (40×) scanning may help to overcome diagnostic challenges due to resolution.

In addition, other studies have shown that there is about a 10% discordance rate between digital whole slide images and glass slide interpretations. However, it is important to note that studies have also demonstrated a 1–5% discordance rate for glass to glass interpretations among randomly selected retrospective quality assurancecases.[2] This study has a comparable 5.7% discordance between the glass reference diagnosis and the glass consultant diagnosis. However, the authors did not thoroughly discuss this. Glass to glass discordance should be used as the gold standard for comparison when evaluating digital pathology. Making a morphologic diagnosis digitally is not going to remove the inherent differences in diagnostic interpretation among pathologists. In addition, there are some elements in the digital format which have not been optimized or even standardized for that matter. Important factors to consider include scan magnification, monitor resolution, monitor size, color calibration of the scan, and calibration of the monitor. In future studies, comparisons of WSI to GSI need to eliminate some of these inherent variables in the digital platform. Quantification and standardization of these factors will facilitate comparison of future studies of WSI. In the coming years, it may even be possible for optimized digital pathology systems to reduce interobserver diagnostic variability by using image analysis algorithms.
